# Digital gene expression profiling of the transcriptional response to *Sclerotinia sclerotiorum* and its antagonistic bacterium *Bacillus amyloliquefaciens* in soybean

**DOI:** 10.3389/fmicb.2022.1025771

**Published:** 2022-11-02

**Authors:** Jianfeng Liu, Xianwen Hu, Hongli He, Xingzheng Zhang, Jinhua Guo, Jing Bai, Yunqing Cheng

**Affiliations:** ^1^Jilin Provincial Key Laboratory of Plant Resource Science and Green Production, Jilin Normal University, Siping, Jilin, China; ^2^College of Food Science and Biology, Hebei University of Science and Technology, Shijiazhuang, Hebei, China

**Keywords:** soybean, dual RNA sequencing, *Sclerotinia sclerotiorum*, *Bacillus amyloliquefaciens*, antagonism

## Abstract

Soybean Sclerotinia stem rot caused by *Sclerotinia sclerotiorum* is a common disease in soybean, and effective biological control is urgently needed. We have previously confirmed that *Bacillus amyloliquefaciens* can effectively antagonize *S. sclerotiorum* in a plate competition experiment and a soybean seedling inoculation experiment. In this study, the mechanisms underlying plant death caused by *S. sclerotiorum* and soybean resistance to *S. sclerotiorum* induced by *B. amyloliquefaciens* were evaluated. The stems of potted soybean seedlings were inoculated with *S. sclerotiorum* (Gm-Ss), *B. amyloliquefaciens* (Gm-Ba), and their combination (Gm-Ba-Ss), using scratch treatments as a control, followed by dual RNA sequencing and bioinformatics analyses. Global gene expression levels in the Gm-Ss treatment were much lower than those in the Gm-Ba, Gm-Ba-Ss, and Gm groups, suggesting that *S. sclerotiorum* strongly inhibited global gene expression in soybean. In a pairwise comparison of Gm-Ss vs. Gm, 19983 differentially expressed genes (DEGs) were identified. Down-regulated DEGs were involved in various KEGG pathways, including ko01110 (biosynthesis of secondary metabolites), ko01100 (metabolic pathways), ko01120 (microbial metabolism in diverse environments), ko00500 (starch and sucrose metabolism), and ko04075 (plant hormone signal transmission), suggesting that *S. sclerotiorum* inoculation had a serious negative effect on soybean metabolism. In Gm-Ba vs. Gm, 13091 DEGs were identified, and these DEGs were significantly enriched in ko03010 (ribosome) and ko03008 (ribosome biogenesis in eucaryotes). Our results suggest that *B. amyloliquefaciens* increases the expression of genes encoding the ribosomal subunit, promotes cell wall biogenesis, and induces systemic resistance. *S. sclerotiorum* strongly inhibited metabolism in soybean, inhibited the synthesis of the cytoskeleton, and induced the up-regulation of programmed death and senescence-related genes *via* an ethylene signal transduction pathway. These results improve our understanding of *S. sclerotiorum-*induced plant death and soybean resistance to *S. sclerotiorum* induced by *B. amyloliquefaciens* and may contribute to the improvement of strategies to avoid yield losses.

## Introduction

*Bacillus amyloliquefaciens* is one of the most important biocontrol strains; it secretes proteins, lipopeptides, and secondary metabolites that inhibit the growth of plant pathogens. Two main kinds of antibacterial substances are secreted by *B. amyloliquefaciens*, i.e., antibacterial substances synthesized by a ribosomal pathway (such as lipopeptides and antibacterial protein β-1,3-glucanase) and fatty substances synthesized by a non-ribosomal pathway (including surfactin, iturin, and fengycin) (Arguelles-Arias et al., 2009; Chen et al., 2009; Arrebola et al., 2010; Salazar et al., 2017). These antibacterial substances can degrade the mycelium of pathogenic fungi (Liao et al., 2016), inhibit mycelial growth and the formation of sclerotia (Chen et al., 2005), and inhibit spore proliferation (Li et al., 2015). Thus, *B. amyloliquefaciens* can antagonize the growth of many plant pathogens, including but not limited to *Fusarium solani* (Kim Y. G. et al., 2015), *Erwinia amylovora* (Xie et al., 2017), *Phytophthora capsica* (Bhusal and Mmbaga, 2020), *Sclerotinia sclerotiorum* (Cheng et al., 2022), *Xanthomonas oryzae* pv. *oryzae* (Xie et al., 2017), *Aeromonas hydrophila* (Li et al., 2021, 2016), and *Fusarium oxysporum* (Kim D. et al., 2015). In addition to inhibiting the growth and reproduction of pathogenic microorganisms, *B. amyloliquefaciens* can induce and strengthen the systemic resistance of plants, thereby conferring protection against pathogenic microorganisms. *B. amyloliquefaciens* FZB42 can promote plant growth and enhance the defense response in *Arabidopsis* (Fan et al., 2012; Chowdhury et al., 2015). This strain can also induce systemic resistance *via* a jasmonic acid-dependent signaling pathway (Xie et al., 2017). *B. amyloliquefaciens* is characterized by strong tolerance and broad antimicrobial activity (Li et al., 2016). It exerts an efficient and broad-spectrum inhibitory effect on the growth and development of pathogenic fungi (Kang et al., 2020). Therefore, probiotic *B. amyloliquefaciens* plays an important role in plant disease control (Zhao, 2021).

*Sclerotinia sclerotiorum* can survive in soil for up to 5–8 years and causes Soybean Sclerotinia stem rot, a major soil-borne disease, restricting soybean production (Clarkson et al., 2003; Zhao et al., 2006; Zhang et al., 2008; Calla et al., 2009). *S. sclerotiorum* mainly infects soybean plants at the seedling, flowering, and pod setting stages, resulting in serious wilting, the decay of leaves and pods, and a sharp decline in yield (Calla et al., 2009). The antagonistic effects of *Bacillus subtilis* (Zhang et al., 2006), mycoviruses (Zhang et al., 2020), *Coniothyrium minitans* (Gao et al., 2006; Mcquilken et al., 2010; Zeng et al., 2012), *Bacillus thuringiensis* (Wang et al., 2020), *Trichoderma asperelloides* (Sumida et al., 2018; de Rezende et al., 2020), *B. amyloliquefaciens* (Chen et al., 2005), and *S. sclerotiorum* hypovirulence-associated DNA virus 1 (Kraberger et al., 2013; Qu et al., 2021) on *S. sclerotiorum* have been studied with the aim of developing an effective biocontrol agent against *S. sclerotiorum.* In a plate assay, the extracellular broth of *B. amyloliquefaciens* could strongly inhibit the growth of *S. sclerotiorum*. After *S. sclerotiorum* was inoculated into the stems of soybean seedlings (by wrapping the cut in plastic cling film for retaining moisture at 20–25°C), the aboveground parts withered completely within 3–5 days; *B. amyloliquefaciens* was not pathogenic to soybean and strongly inhibited the growth of *S. sclerotiorum*. A transcriptome analysis further showed that *B. amyloliquefaciens* could strongly inhibit gene expression in *S. sclerotiorum* inoculated in soybean seedlings. *B. amyloliquefaciens* inhibits *S. sclerotiorum* growth by altering the expression of ribosomal genes, resulting in the inhibition of protein synthesis in *S. sclerotiorum*. These findings provided new insights into the mechanisms by which *B. amyloliquefaciens* antagonizes pathogenic microorganisms. However, there is no germplasm that can resist *S. sclerotiorum* completely in soybean. Williams 82 is a soybean variety sensitive to *S. sclerotiorum* (Calla et al., 2009). In the presence of *B. amyloliquefaciens*, our previous studies have confirmed that Williams 82 seedlings are completely resistant to *S. sclerotiorum*. There are two potential explanations for this resistance. It is possible that the antibacterial components of *B. amylolyticus* can inhibit the growth of *S. sclerotiorum*. Alternatively, *B. amyloliquefaciens* can alter the expression of soybean resistance genes and thereby enhance resistance to *S. sclerotiorum*. A series of pathogenic factors of *S. sclerotiorum* have been identified, including oxalic acid (Kim et al., 2008), cell wall-degrading enzymes (Pan et al., 2015), and polygalacturonases (Fraissinet-Tachet et al., 1995). However, genes involved in soybean resistance to *S. sclerotiorum* and the pathogenic mechanism are still largely unresolved.

To clarify the interactions between soybean, *B. amyloliquefaciens*, and *S. sclerotiorum*, we used dual RNA-seq technology to analyze gene expression changes in soybean seedling after inoculation with *S. sclerotiorum* alone, *B. amyloliquefaciens* alone, or their mixture. Our results provide insight into the pathogenic mechanism of *S. sclerotiorum* on soybean and genes involved in soybean resistance to *S. sclerotiorum*.

## Materials and methods

### Experimental microbial strains

The experimental microorganisms used in present study was from China General Microbiological Culture Collection Center (CGMCC), including *S. sclerotiorum* (NO 3.7083) and our patented strain *B. amyloliquefaciens* (NO 20507). Before inoculation in soybean seedling, these strains were cultured and activated in potato dextrose agar (PDA) medium at 28°C.

### Inoculation treatments of soybean seedling stems

**FIGURE 1:**
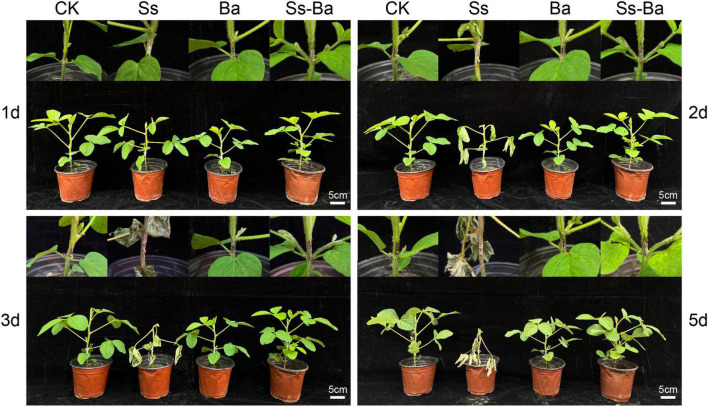
Potted *G. max* seedling inoculated with *B. amyloliquefaciens*, *S. sclerotiorum*, and their mixture. Ba, Ss, and Ss-Ba indicated that the stem of soybean seedling was inoculated with *B. amyloliquefaciens*, *S. sclerotiorum*, and both microorganisms, respectively. Ck, scratch treatments. The words of 1, 2, 3, and 5 d on the left and right of the picture indicate the days after inoculation.

Seeds were sown in plastic pots and cultured in an artificial climate chamber. Then, 40 seedlings of *Glycine max* cultivar Williams 82 (w82) with uniform growth were used for inoculation experiments. At the V4 stage described by Fehr and Caviness (1977), the stems of soybean seedlings were inoculated with *S. sclerotiorum* (referred to as Gm-Ss), *B. amyloliquefaciens* (Gm-Ba), and a mixture of *S. sclerotiorum* and *B. amyloliquefaciens* (Gm-Ba-Ss), and scratch treatments were used as the control (Gm). The site of inoculation or scratch was in the middle of the two lowest nodes. On the second day after inoculation, seedling leaves of Gm-Ss began to wilt and showed obvious symptoms. On days 3 and 5 after inoculation, seedling lodging of Gm-Ss was obvious, and seedlings of the other three treatments showed no obvious symptoms (Figure 1). Based on these results, a 2 cm segment of the stem surrounding the inoculation site was obtained for RNA extraction. There were three biological replicates in each treatment, and three soybean stem segments were included in each replicate. The biological samples used in this study were the same as those used in a previous study (Cheng et al., 2022), and the cultivation of soybean seedlings, preparation of inoculated microorganisms, and inoculation methods have been described in detail (Cheng et al., 2022).

### cDNA library preparation and sequencing

To explore the interactions between soybean and *B. amyloliquefaciens* and between soybean and *S. sclerotiorum*, 12 digital gene expression (DGE) profiling libraries were constructed for Gm-Ss, Gm-Ba, Gm-Ba-Ss, and Gm (three replicates each). The RNA EasySpin Isolation System (Aidlab Biotech, Beijing, China) was used to perform total RNA extraction from 12 biological samples. Electrophoresis on a 1% agarose gel was used to evaluate RNA degradation and contamination of total RNA. A NanoPhotometer (IMPLEN, CA, USA) was used to assess the purity of RNA. A total amount of 2 μg of RNA was pretreated using the RNA Nano 6000 Assay Kit (Agilent Technologies, CA, USA) following the manufacturer’s instructions, followed by an assessment of RNA integrity using the Bioanalyzer 2100 system (Agilent Technologies, CA, USA). To obtain sequencing libraries, the RNA samples were pretreated using the NEBNext Ultra RNA Library Prep Kit for Illumina (Illumina, Inc., USA) following the manufacturer’s instructions. The main processing flow is as follows. First, the Ribo-Zero rRNA Removal Kit was used to eliminate rRNA to obtain purified mRNA, followed by fragmentation using divalent cations in NEBNext First Strand Synthesis Reaction Buffer under high-temperature conditions. RNase H and random hexamer primers were used to synthesize the first-strand cDNA, followed by second-strand cDNA synthesis using dNTPs, DNA polymerase I, and RNase H. AMPure XP beads were used to purify the cDNA libraries. The libraries were subjected to elution with EB buffer, terminal repairing, A-tailing, and adapter addition, in sequence, and agarose gel electrophoresis was performed to retrieve the products. Second-strand cDNA was digested with UNG followed by PCR. Then, preliminary quantification of cDNA libraries was carried out using Qubit4.0, and the libraries were diluted to 1.0 ng/μL. Finally, the quality of the cDNA library was evaluated using the Bioanalyzer 2100 system (Agilent Technologies, CA, USA). The Illumina NovaSeq6000 platform was used to perform PE150 (double-terminal 150 bp) sequencing of the 12 cDNA libraries.

### Bioinformatics analysis of dual RNA-seq data

To obtain clean reads, cutadapt (v2.7)^1^ was used to filter reads including adapters, reads with quality value < Q20, reads containing N > 10%, and reads with length < 25 bp. Samples from Gm-Ss, Gm-Ba, and Gm-Ba-Ss treatments included RNA from *S. sclerotiorum* and *B. amyloliquefaciens* in addition to soybean RNA. Reads from *S. sclerotiorum* or *B. amyloliquefaciens* were filtered out to avoid background interference, and only sequences from *G. max* were retained for further bioinformatics analyses. To remove reads from *S. sclerotiorum* or *B. amyloliquefaciens* in the Gm-Ba-Ss treatment, the data were aligned to the reference genome of *B. amyloliquefaciens*^2^ and the matched sequences were removed. Then, the remaining data were aligned to the reference genome of *S. sclerotiorum*^3^ and the matched sequences were removed. Finally, the remaining data were aligned to the reference genome of *G. max*^4^ and the matched sequences belonging to *G. max* were subjected to further bioinformatics analyses. HISAT2 was used to generate sequence alignments and for sequence removal (Kim Y. G. et al., 2015). The remaining mapped reads were assembled using Stringtie (v1.3.3b) (Pertea et al., 2015). For transcript annotation, the soybean coding sequences (CDS) and corresponding amino acid sequences were predicted using TransDecoder (r201311110). Then, transcript CDS and amino acid sequences were aligned to the Pfam (protein family) database using Hmmer (v3.3.2) to obtain protein domain classification information. Finally, the transcripts were searched against NR, string, SwissProt, and KEGG to obtain annotation information using diamond (v2.0.8). The COG and GO annotations were parsed from the string and SwissProt annotations, respectively. According to the alignment results obtained using HISAT2, Stringtie (v1.3.3b) was used to calculate the FPKM (fragments per kilobase of transcript per million fragments mapped) value of each gene (Pertea et al., 2015). Differentially expressed genes (DEGs) were identified using DESeq2 (Love et al., 2014), setting | log_2_FoldChange| > 1 and a false discovery rate (FDR) < 0.05 as thresholds. FoldChange in the formula is the ratio of FPKM values in the pairwise comparison, and FDR is the adjusted *p*-value. Heatmaps showing DEGs FPKM values were generated using HeatMap Illustrator in TBtools (v1.098726) by normalized scale method. DEGs were evaluated by KEGG pathway and GO enrichment analyses, as previously described (Tan and Lenhard, 2016), setting *p* < 0.05 and FDR < 0.05 as thresholds for significant enrichment. The position and direction of change (up-regulation or down-regulation) of DEGs in the metabolic pathway were visually analyzed using iPath3.0^5^ (Letunic et al., 2008).

### qRT-PCR verification

To verify the reliability of the dual RNA-seq data, six genes were randomly selected for further qRT-PCR, and soybean *Actin* was used as an internal reference gene. Total RNA was extracted from 12 soybean stem samples for qRT-PCR using the RNA EasySpin Isolation System (Aidlab Biotech, Beijing, China), following the manufacturer’s instructions. The samples were the same as those used for the dual RNA-seq analysis. PCR primers were designed using Primer Premier 5. The primer sequence information is provided in Supplementary Table 1. The parameters for PCR were set as described previously (Liu et al., 2021). The 2^–ΔΔCt^ method was used to calculate the expression differences (Schmittgen and Livak, 2008).

## Results

### RNA-seq, data quality control, and read alignment

Dual RNA-seq of 12 biological samples from four treatments generated 1,197.74 million raw reads. In total, 1,197.02 million clean reads were obtained after data filtering, covering a length of 169,513,862,836 bp with an average read length of 141.61 bp. The average Q20 value of all reads was 97.73% (Supplementary Table 2). The clean data were aligned to the soybean genome for transcript annotation. In four treatments, and the mapped read ratios for Gm-Ba, Gm-Ba-Ss, and Gm ranged from 93.21 to 96.39%, while those of Gm-Ss ranged from 24.43 to 25.70%, suggesting that rapid *S. sclerotiorum* reproduction resulted in a dramatic decrease in soybean RNA levels in Gm-Ss treatments. For Gm-Ba, Gm-Ba-Ss, and Gm, 89.63–93.76% clean reads could be uniquely mapped to the soybean reference genome, which was much higher than values of 21.52–22.66% of uniquely mapped clean reads obtained in the Gm-Ss treatments (Supplementary Table 3). We annotated all genes using six databases, namely NR, SwissProt, Pfam, STRING, GO, and KEGG and obtained annotation information for 52,872 genes (Supplementary Table 4).

### Comparison of global gene expression levels

**FIGURE 2:**
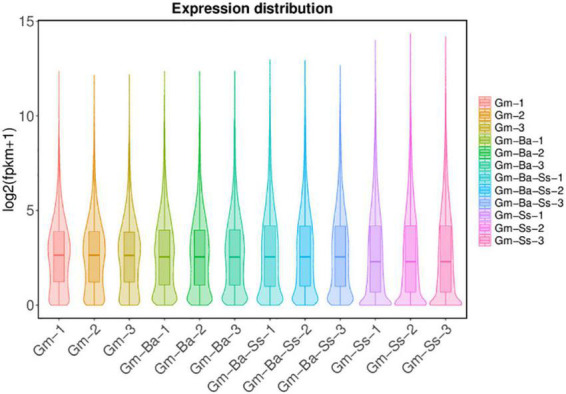
Violin diagram of gene expression. Gm-Ba, *G. max* seedling inoculated with *B. amyloliquefaciens*; Gm-Ss, *G. max* seedling inoculated with *S. sclerotiorum*; Gm-Ba-Ss, *G. max* seedling inoculated with *B. amyloliquefaciens* and *S. sclerotiorum*; Gm, control, scratch treatment.

For the Gm, Gm-Ba, and Gm-Ba-Ss treatments, similar numbers of genes were expressed (i.e., 43694, 44446, and 44574 genes, respectively) (Supplementary Table 5). In Gm-Ss, 38556 genes were expressed, which was fewer than estimates in the other three treatments (Supplementary Table 5). To compare gene expression differences at the global level, we drew a violin map representing the transcript levels in each sample (Figure 1). The global gene expression levels of the three biological samples from the Gm-Ss treatment were much lower than those of the Gm-Ba, Gm-Ba-Ss, and Gm treatments (Figure 2), suggesting that *S. sclerotiorum* inhibits global gene expression in soybean.

### Differentially expressed genes in paired comparisons

**FIGURE 3:**
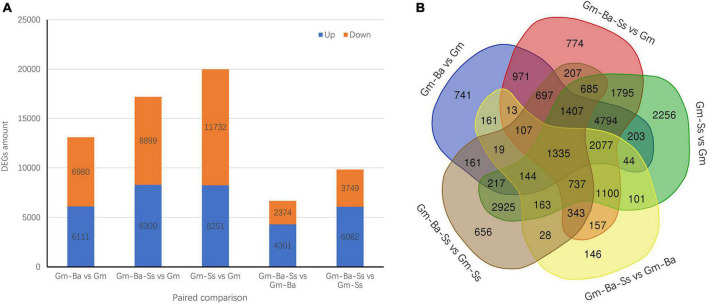
Differentially expressed genes (DEGs) in five paired comparisons. **(A)** The DEGs amount from five paired comparisons. **(B)** Venn diagrams of DEGs from five paired comparisons. Gm-Ba, *G. max* seedling inoculated with *B. amyloliquefaciens*; Gm-Ss, *G. max* seedling inoculated with *S. sclerotiorum*; Gm-Ba-Ss, *G. max* seedling inoculated with *B. amyloliquefaciens* and *S. sclerotiorum*; Gm, control, scratch treatment.

We identified all DEGs in five pairwise comparisons (listed in Supplementary Tables 6–10). In Gm-Ba vs. Gm, Gm-Ba-Ss vs. Gm, and Gm-Ss vs. Gm, 13091, 17199, and 19983 DEGs were identified, respectively (Figure 3A). The most DEGs were identified in Gm-Ss vs. Gm, consistent with the obvious morphological changes caused by inoculation with *S. sclerotiorum* alone. In addition, 11732 down-regulated genes and 8251 up-regulated genes were identified in Gm-Ss vs. Gm, and the number of down-regulated genes was 1.42 times the number of up-regulated genes, indicating that *S. sclerotiorum* inoculation significantly inhibited the expression of soybean genes, consistent with the results shown in Figure 2. In Gm-Ba-Ss vs. Gm-Ba and Gm-Ba-Ss vs. Gm-Ss, 6675 and 9831 DEGs were identified, respectively (Figure 3A). The number of up-regulated genes was much higher than the number of down-regulated genes. This showed that compared with *B. amyloliquefaciens* and *S. sclerotiorum* inoculation alone, mixed inoculation with *B. amyloliquefaciens* and *S. sclerotiorum* was more conducive to promoting the expression of soybean genes.

Differentially expressed genes in the five pairwise comparisons were evaluated to identify unique and common DEGs, as visualized using Venn diagrams. In total, 741, 774, 2256, 146, and 656 unique DEGs were identified in Gm-Ba vs. Gm, Gm-Ba-Ss vs. Gm, Gm-Ss vs. Gm, Gm-Ba-Ss vs. Gm-Ba, and Gm-Ba-Ss vs. Gm-Ss, respectively (Figure 3B). There were far more unique DEGs in Gm-Ss vs. Gm than in the other four pairwise comparisons. Furthermore, 1335 common DEGs in the five paired comparisons were identified (Figure 3B).

### Gene ontology enrichment of differentially expressed genes from different paired comparisons

To explore the possible functions of DEGs in different paired comparisons, a GO enrichment analysis was performed. The significantly enriched GO terms in the five comparisons are listed in Supplementary Tables 11–15. All DEGs were assigned to terms in three GO categories (i.e., cellular components, biological processes, and molecular functions).

**FIGURE 4:**
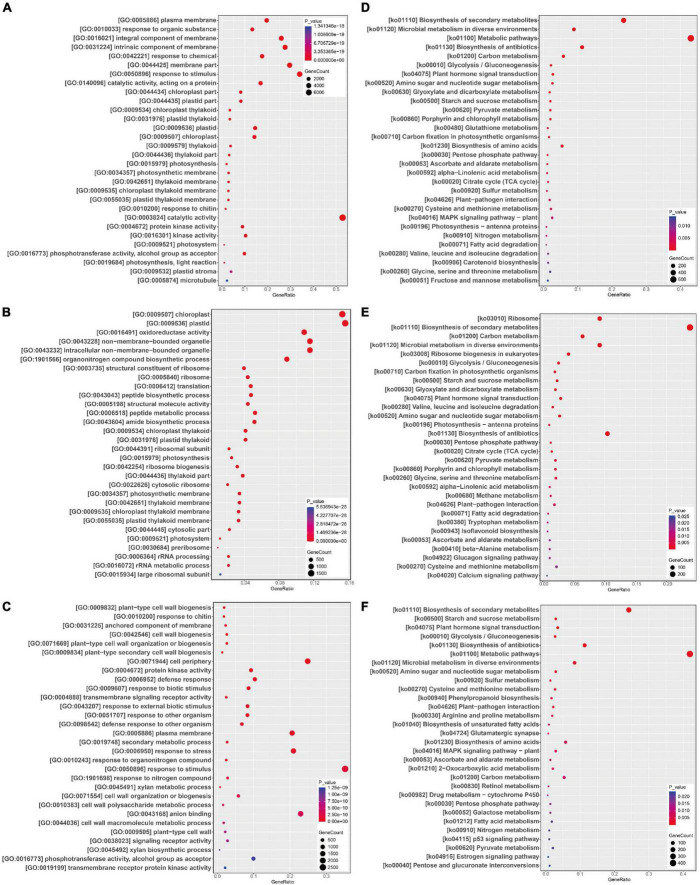
GO functional and KEGG pathway enrichment analysis of differentially expressed genes (DEGs). DEGs GO functional and KEGG pathway enrichment analysis in paired comparison of Gm-Ss vs. Gm **(A,D)**, Gm-Ba vs. Gm **(B,E)**, and Gm-Ba-Ss vs. Gm-Ss **(C,F)**. The abscissa GeneRatio represents the proportion of DEGs belonging to a GO term and KEGG pathways in all DEGs; The larger the bubble, the more DEGs; Color indicates *P*-value, representing the significance of enrichment. The darker the color, the more significantly enriched the GO term and KEGG pathways. The right color gradient indicates *P*-value.

For Gm-Ss vs. Gm, the most significantly enriched GO terms in the biological process category were GO:0010033 (response to organic substance) and GO:0042221 (response to chemical), followed by GO:0050896 (response to stimulus), GO:0015979 (photosynthesis), and GO:0010200 (response to chitin) (Figure 4A). The most significantly enriched GO terms in the cellular components category were GO:0005886 (plasma membrane), GO:0016021 (integral component of membrane), and GO:0031224 (intrinsic component of membrane) (Figure 4A); the most significantly enriched GO terms in the molecular functions category were GO:0140096 (catalytic activity, acting on a protein) and GO:0003824 (catalytic activity), followed by GO:0004672 (protein kinase activity), GO:0016301 (kinase activity), and GO:0016773 (phosphotransferase activity, alcohol group as acceptor) (Figure 4A).

For Gm-Ba vs. Gm, the most significantly enriched GO terms in the biological process category were GO:1901566 (organonitrogen compound biosynthetic process) and GO:0006412 (translation), followed by GO:0043043 (peptide biosynthetic process), GO:0006518 (peptide metabolic process), and GO:0043604 (amide biosynthetic process) (Figure 4B). The most significantly enriched GO terms in the cellular components category were GO:0009507 (chloroplast), GO:0009536 (plastid), GO:0043228 (non-membrane-bounded organelle), GO:0043232 (intracellular non-membrane-bounded organelle), and GO:0005840 (ribosome) (Figure 4B). The most significantly enriched GO terms in the molecular functions category were GO:0016491 (oxidoreductase activity), GO:0003735 (structural constituent of ribosome), GO:0005198 (structural molecule activity), GO:0019843 (rRNA binding), and GO:0030515 (snoRNA binding) (Figure 4B).

For Gm-Ba-Ss vs. Gm-Ss, the most significantly enriched GO terms in the biological process category were GO:0009832 (plant-type cell wall biogenesis), GO:0010200 (response to chitin), GO:0042546 (cell wall biogenesis), GO:0071669 (plant-type cell wall organization or biogenesis), and GO:0009834 (plant-type secondary cell wall biogenesis) (Figure 4C). The most significantly enriched GO terms in the cellular components category were GO:0031225 (anchored component of membrane), GO:0071944 (cell periphery), GO:0005886 (plasma membrane), GO:0009505 (plant-type cell wall), and GO:0030686 (90S preribosome) (Figure 4C). The most significantly enriched GO terms in the molecular functions category were GO:0004672 (protein kinase activity), GO:0004888 (transmembrane signaling receptor activity), GO:0043168 (anion binding), GO:0038023 (signaling receptor activity), and GO:0016773 (phosphotransferase activity, alcohol group as acceptor) (Figure 4C).

### Kyoto encyclopedia of genes and genomes enrichment of differentially expressed genes from different paired comparisons

All DEGs in the five comparisons were evaluated by a KEGG pathway enrichment analysis. The significantly enriched KEGG pathways are listed in Supplementary Tables 16–20. *S. sclerotiorum* had an important impact on soybean metabolism. In Gm-Ss vs. Gm, the most significantly enriched KEGG pathway was ko01110 (Biosynthesis of secondary metabolites), followed by ko01120 (Microbial metabolism in diverse environments), ko01100 (Metabolic pathways), ko01130 (Biosynthesis of antibiotics), ko01200 (Carbon metabolism), and ko00010 (Glycolysis/Gluconeogenesis). *B. amyloliquefaciens* had an important impact on Genetic Information Processing and metabolism in soybean (Figure 4D). In Gm-Ba vs. Gm, the most significantly enriched KEGG pathway was ko03010 (Ribosome), followed by ko01110 (Biosynthesis of secondary metabolites), ko01200 (Carbon metabolism), ko01120 (Microbial metabolism in diverse environments), and ko03008 (Ribosome biogenesis in eukaryotes) (Figure 4E). *S. sclerotiorum* inoculation alone caused the quick death of soybean seedlings, and its mixture with *B. amyloliquefaciens* did not produce obvious symptoms. In Gm-Ba-Ss vs. Gm-Ss, the most significantly enriched KEGG pathway was ko01110 (Biosynthesis of secondary metabolites), followed by ko00500 (Starch and sucrose metabolism), ko04075 (Plant hormone signal transduction), ko00010 (Glycolysis/Gluconeogenesis), and ko01130 (Biosynthesis of antibiotics) (Figure 4F).

**FIGURE 5:**
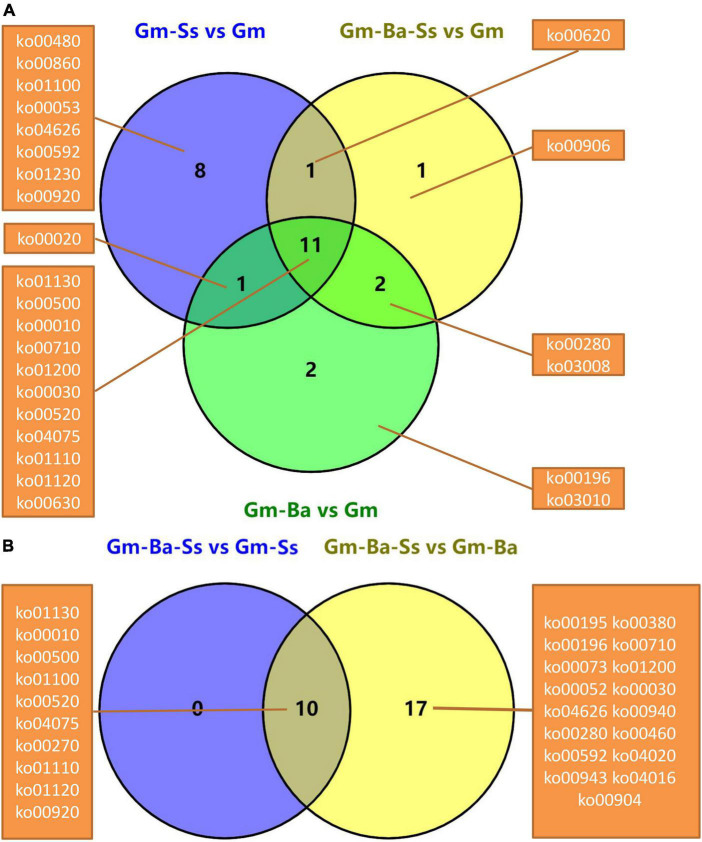
Venn diagrams of significantly enriched KEGG pathway. **(A)** Venn diagrams of significantly enriched KEGG pathway from three pairwise comparisons of Gm-Ba vs. Gm, Gm-Ba-Ss vs. Gm, Gm-Ss vs. Gm. **(B)** Venn diagrams of significantly enriched KEGG pathway from two pairwise comparisons of Gm-Ba-Ss vs. Gm-Ss and Gm-Ba-Ss vs. Gm-Ba.

Unique and common significantly enriched KEGG pathways in the five comparisons were evaluated based on Venn diagrams. For Gm-Ba vs. Gm, Gm-Ba-Ss vs. Gm, and Gm-Ss vs. Gm, there were 26 significantly enriched KEGG pathways (Figure 5A). There were eight unique significantly enriched KEGG pathways for DEGs in Gm-Ss vs. Gm, more than two for Gm-Ba vs. Gm and one for Gm-Ba-Ss vs. Gm. DEGs in these three paired comparisons were involved in 11 common pathways, accounting for 42.31% of all significantly enriched KEGG pathways. For DEGs in Gm-Ba-Ss vs. Gm-Ss and Gm-Ba-Ss vs. Gm-Ba, there were 17 significantly enriched KEGG pathways (Figure 5B). Gm-Ba-Ss vs. Gm-Ss and Gm-Ba-Ss vs. Gm-Ba shared 10 common significantly enriched KEGG pathways. For Gm-Ba-Ss vs. Gm-Ss, there were no unique significantly enriched KEGG pathways. For Gm-Ba-Ss vs. Gm-Ss, there were 17 unique significantly enriched KEGG pathways. Collectively, the above results showed that the effect of *S. sclerotiorum* on soybean gene expression was offset by *B. amyloliquefaciens.*

### Important differentially expressed genes involved in plant-microbiome interactions and signaling pathways

**FIGURE 6:**
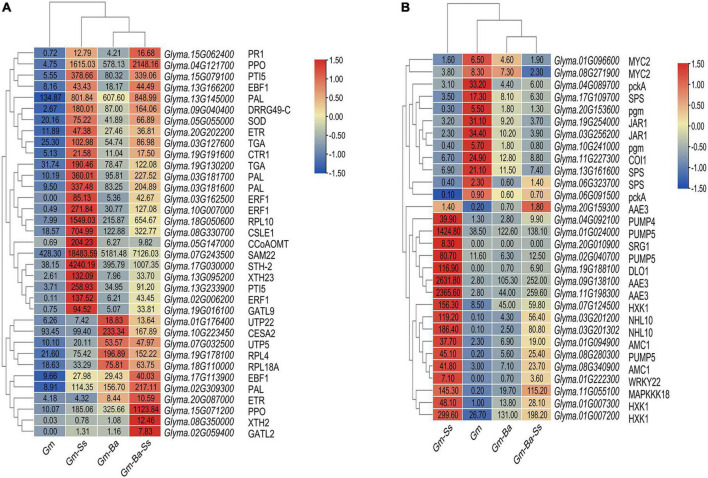
Heatmaps of soybean DEGs. **(A)** DEGs possibly related to the improvement of soybean resistance by *B. amyloliquefaciens*. **(B)** DEGs possibly related to soybean seedling senescence by *S. sclerotiorum*. The number in each heatmap box represents the FPKM value of each gene. AAE3, Oxalate-CoA ligase; *CCoAOMT*, caffeoyl-CoA 3-*O*-methyltransferase; CESA2, Cellulose synthase A catalytic subunit 2; COI1, coronatine-insensitive protein 1; *CSLE1*, Cellulose synthase-like protein E1; CTR1, serine/threonine-protein kinase CTR1; DLO1, DMR6-LIKE OXYGENASE 1; DRRG49-C, Disease resistance response protein DRRG49-C; EBF1, EIN3-binding F-box protein; ERF1, ethylene-responsive transcription factor 1; ETR, ethylene receptor; GATL2, galacturonosyltransferase-like 2; GATL9, galacturonosyltransferase-like 9; HXK1, Hexokinase-1; JAR1, jasmonic acid-amino synthetase; MAPKKK18, mitogen-activated protein kinase kinase kinase 18; AMC1, Metacaspase-1; MYC2, transcription factor MYC2; NHL10, NDR1/HIN1-like protein 10; PAL, phenylalanine ammonia lyase; pckA, phosphoenolpyruvate carboxykinase (ATP); pgm, phosphoglucomutase; PPO, polyphenol oxidase; PR1, pathogenesis related protein 1; PTI5, Pathogenesis-related genes transcriptional activator PTI5; PUMP, mitochondrial uncoupling protein; RPL10, ribosomal protein L10; RPL18A, ribosomal protein L18a; RPL4, ribosomal protein L4; SAM22, Stress-induced protein SAM22; SOD, Superoxide dismutase; SPS, Sucrose-phosphate synthase; SRG1, protein SRG1; STH-2,Pathogenesis-related protein STH-2; TGA, transcription factor TGA; UTP22, U3 small nucleolar RNA-associated protein 22; UTP5, U3 small nucleolar RNA-associated protein 5; WRKY22, WRKY transcription factor 22; XTH, Xyloglucan endotransglucosylase/hydrolase protein.

Ethylene and salicylic acid (SA) are closely related to the formation of systemic acquired resistance (SAR) in plants (Chen et al., 2020). In this study, a number of DEGs encoding important members of the ethylene and SA signal transduction pathways were identified (Figure 6A). DEGs encoding members of SA signaling pathways included TGA (e.g., Glyma.19G130200, Glyma.03G127600) and PRs (e.g., Glyma.15G062400, Glyma.09G040400, Glyma.15G079100, Glyma.07G243500, Glyma.17G030000, Glyma.13G233900). The DEGs encoding members of ethylene signaling pathways included ETR (Glyma.20G087000 and Glyma.20G202200), CTR1 (Glyma.19G191600), and EBF1 (Glyma.13G166200, Glyma.17G113900, Glyma.03G162500, Glyma.10G007000, and Glyma.02G006200). In addition, a set of DEGs encoding SAR-related genes including *PAL* and *PPO* were expected to be involved in plant-microbiome interactions (see Figure 6A for the most highly expressed genes). Finally, DEGs encoding *CSLE1*, *CESA2*, *XTH2*, *XTH23*, *CCoAOMT*, *GATL2*, and *GATL9* are known to be involved in the biosynthesis of cell well components, such as cellulose, lignin, and pectin. Among these, *TGA*, *DRRG49-C*, *PTI5*, *CTR1*, *PAL*, *SOD*, *CSLE1*, *XTH23*, and *CCoAOMT* had much higher FPKM values in the Gm-Ss than those in the Gm-Ba-Ss, Gm-Ba, and Gm treatment groups, suggesting that their expression was promoted by Gm-Ss treatment.

**FIGURE 7:**
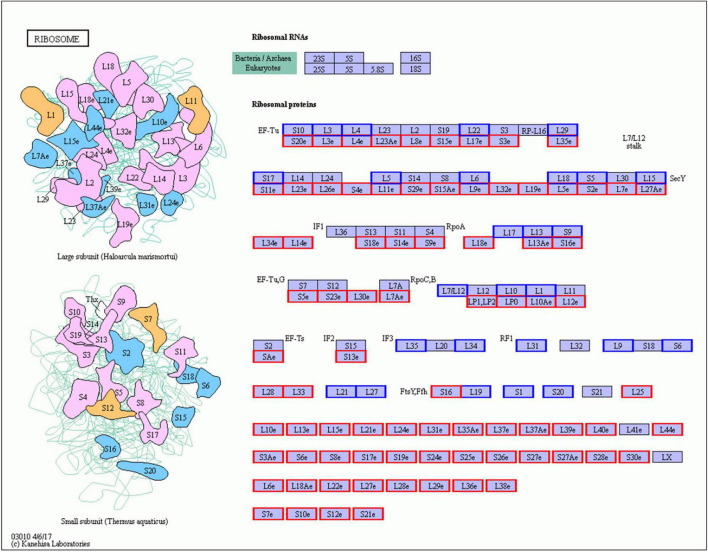
Up- and down-regulated genes of ko03010 KEGG pathway in paired comparison of Gm-Ba vs. Gm. ko03010, ribosome KEGG pathway. The genes with red or blue border in the figure were differentially expressed genes, where red and blue represents up- and down-regulated genes, respectively.

**FIGURE 8:**
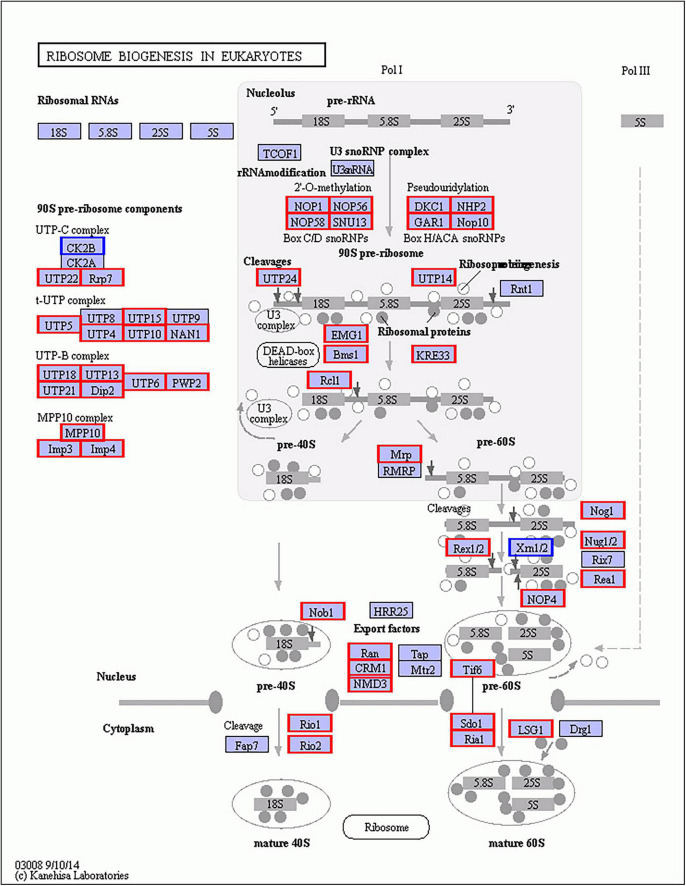
Up- and down-regulated genes of ko03008 KEGG pathway of in paired comparison of Gm-Ba vs. Gm. ko03008, ribosome biogenesis in eucaryotes. The genes with red or blue border in the figure were differentially expressed genes, where red and blue represents up- and down-regulated genes, respectively.

**FIGURE 9:**
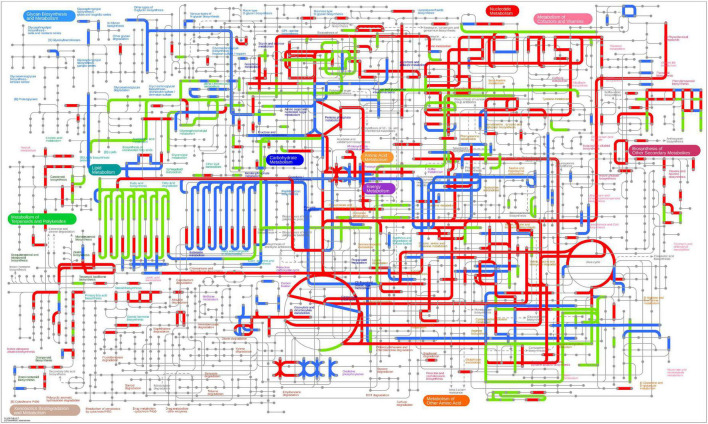
Distribution of DEGs on iPath integration metabolic pathway in paired comparison of Gm-Ba-Ss vs. Gm. The red line represents the pathway affected by up-regulated genes, the blue line represents the pathway affected by down-regulated genes, and the yellow line represents the pathway affected by both up-regulated and down-regulated genes.

In Gm-Ba vs. Gm, DEGs were significantly enriched in ko03010 (ribosome) and ko03008 (ribosome biogenesis in eucaryotes), and most genes involved in these pathways were up-regulated (Figure 6A; Supplementary Table 17). These DEGs had much higher FPKM values in the Gm-Ba and Gm-Ba-Ss than those in the Gm-Ss and Gm treatment groups. In ko03010, the up-regulated genes encoded small subunit ribosomal proteins S20e/S18e/SAe/S13e and large subunit ribosomal proteins L3e/L34e/L14e/L10e, etc. (Figure 7). Many important members of the ko03008 pathway were up-regulated, including the rRNA gene for 2′-*O*-methyltransferase fibrillarin, nucleolar protein 56, U3 small nucleolar RNA-associated protein 24, 60S ribosomal export protein NMD3, and ribosome assembly protein 1 (Figure 8). Finally, in Gm-Ba-Ss vs. Gm-Ss, more metabolic pathways tended to be activated in an iPath analysis (Figure 9). The results suggest that *B. amyloliquefaciens* could activate ribosome and ribosome biogenesis pathways and soybean metabolism, which may promote soybean resistance against *S. sclerotiorum.*

The FPKM value of DEGs which were possibly related to soybean seedling senescence by *S. sclerotiorum* were showed in Figure 6B. Three DEGs encoding oxalyl-CoA synthetase were identified, two of which were highly up-regulated in response to Gm-Ss. In addition, four DEGs encoding PUMP4/5, which regulates dicarboxylic acid transmembrane transporter activity, were identified and were most highly expressed in the Gm-Ss treatment (Figure 6B). In Gm-Ss vs. Gm, ethylene and SA signal transduction tended to be activated by the up-regulation of vital DEGs encoding TGA, PR1, ETR, CTR1, and EBF1 (Figure 6A), while jasmonic acid (JA) signal transduction tended to be inactivated by the down-regulation of JAR1, COI1, and MYC2 (Figure 6B). In our study, 117 up-regulated DEGs related to senescence and 43 up-regulated DEGs related to programmed cell death were identified. Among these, the most highly up-regulated DEGs involved in the regulation of senescence included DLO1, NHL10, SRG1, WRKY22, and MAPKKK18, and the most highly up-regulated DEGs involved in the regulation of programmed cell death included HXK1 and AMC1. The activation of important components of ethylene signal transduction pathways by *S. sclerotiorum* may further activate genes related to senescence and programmed death, leading to cell collapse and disintegration.

### Verification of gene expression changes using qRT-PCR

**FIGURE 10:**
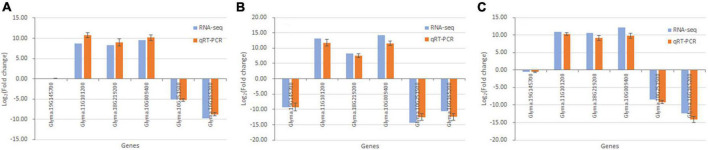
Validation of dual RNA sequencing data using qRT-PCR. **(A)** Six randomly chosen genes in paired comparison of Gm-Ba vs. Gm. **(B)** Six randomly chosen genes in paired comparison of Gm-Ss vs. Gm. **(C)** Six randomly chosen genes in paired comparison of Gm-Ba-Ss vs. Gm.

Six DEGs in the Gm-Ba vs. Gm, Gm-Ss vs. Gm, and Gm-Ba-Ss vs. Gm comparisons were randomly chosen for validation by qRT-PCR. The log_2_(Fold Change) values measured by qRT-PCR and dual RNA-seq are shown in Figure 10. In general, the expression differences obtained by qRT-PCR were consistent with those acquired by dual RNA-seq, indicating that the results of dual RNA-seq were generally reliable.

## Discussion

### *Bacillus amyloliquefaciens* induced systemic acquired resistance of soybean seedlings to *Sclerotinia sclerotiorum via* the activation of ribosome biogenesis

Plants are infected by many pathogenic microorganisms during growth and development. They have evolved two immune defense mechanisms: pathogen-associated molecular pattern (PAMP)-triggered immunity (PTI) (Chisholm et al., 2006) and effector-triggered immunity (ETI) (Jones and Dangl, 2006). Both PTI and ETI can trigger hypersensitive responses in local tissues and SAR in distal tissues (Thomma et al., 2011). SAR can be activated by an elevated concentration of SA and beneficial microbes, such as *Bacillus* spp. (termed induced systemic resistance, ISR) (Lugtenberg and Kamilova, 2009; Chen et al., 2020). The metabolites of *B. amyloliquefaciens* can be used to control root, leaf, and post-harvest diseases of major cash crops, such as sugarcane, tobacco, and tomato (Liu et al., 2020; Tian et al., 2021; Zhao, 2021). *B. amyloliquefaciens* can promote crop growth or inhibit pathogens by producing and secreting secondary metabolites (Salazar et al., 2017). Various products of *B. amyloliquefaciens* have established antagonistic effects, including polyenes, lipopeptides, amino acids, nucleic acids, polyketides, and antibacterial proteins (Arguelles-Arias et al., 2009; Chen et al., 2009; Salazar et al., 2017). Disease resistance mainly involves cell structure destruction of the pathogenic bacteria, triggering SAR and inhibiting the growth of pathogenic bacteria (Liao et al., 2016). Surfactin and fengycin secreted by *B. amyloliquefaciens* can induce SAR in lettuce (Chowdhury et al., 2015). Seedling roots of *Oryza sativa* 9311 were soaked in a *B. amyloliquefaciens* FZB42 suspension for 4 h, and 379 DEGs in roots and 719 in leaves were identified by RNA-seq (Xie et al., 2017), suggesting that FZB42 improves plant pathogen resistance by regulating the expression of pathogenesis-related genes and other defense genes as well as by influencing fundamental metabolic pathways (Xie et al., 2017). *B. amyloliquefaciens* can activate SAR to pathogenic microorganisms causing bacterial pustule through increasing phenols, peroxidase, and 1,3-b-glucanase in soybean plants (Buensanteai et al., 2007; Prathuangwong and Buensanteai, 2007). *B. amyloliquefaciens* FZB42 inoculation suppresses miR846 expression to induce Arabidopsis ISR *via* a JA-dependent signaling pathway (Xie et al., 2018). Ethylene plays an important role in the regulation of broad-spectrum resistance of plants to pathogens (Helliwell et al., 2013, 2016). In *Arabidopsis thaliana*, ISR depends on the phytohormones SA and ethylene (Pozo et al., 2008). However, the signaling factors used by *B. amyloliquefaciens* to initiate ISR are unclear.

Salicylic acid is an essential mediator in the SAR signal transduction pathway. Many SAR genes are encoded by pathogenesis-related proteins (PRs) (Zhou et al., 2021). PRs often exert antifungal activity and are thought to be related to the induction of resistance. In this study, the KEGG pathway ko04075 was a significantly enriched in Gm-Ba vs. Gm, and TGA and PR1, important members of the SA signal transduction pathway, were up-regulated, which may lead to the activation this pathway. Consistent with this, various DEGs encoding PRs were also up-regulated, including *DRRG49-C*, *SAM22*, *STH-2*, and *PTI5* (Figure 6A). In addition, the Gm-Ba treatment activated the ethylene signaling pathway by the up-regulation of *ETR*, *CTR1*, and *ERF1* (Figure 6A). PAL can cause the production of plant antitoxins and phenolic compounds, and PPO is positively related to plant resistance. In this study, we found a set of up-regulated genes encoding PAL and PPO (see Figure 6A for the most highly expressed genes). However, in the Gm-Ss treatment, most DEGs related to the SA signal transduction pathway, ethylene signaling pathway, and plant antitoxins and phenolic compounds biosynthesis had much higher FPKM values than those in the Gm-Ba-Ss, Gm-Ba, and Gm treatments, indicating that the increase of FPKM values relative to SAR in the soybean may be only a stress response to injury. *S. sclerotiorum* led to the rapid death of soybean seedlings after inoculation. The greater the damage, the higher the FPKM value. Therefore, we believe that the biocontrol mechanism of *B. amyloliquefaciens* is relatively complex. For low virulent pathogenic microorganisms, improving the systemic resistance of plants *via* the activation of SA and ethylene signaling transduction pathways by *B. amyloliquefaciens* may be sufficient. For highly virulent pathogenic microorganisms, such as *S. sclerotiorum*, the antibacterial components produced by *B. amyloliquefaciens* should play a major role in resistance, while the improvement of plant SAR by *B. amyloliquefaciens* may play a less important role.

The inhibition of ribosomal gene biosynthesis by *B. amyloliquefaciens* represents a new mechanism underlying *B. amyloliquefaciens* biocontrol (Cheng et al., 2022). Interestingly, *B. amyloliquefaciens* also has an important impact on ribosome biosynthesis in the soybean. The DEGs in ko03010 (ribosome) and ko03008 (ribosome biogenesis in eucaryotes) were involved in rRNA modification, pre-rRNA cleavage in the nucleoplasm, the export of pre-ribosome from the nucleoplasm to cytoplasm, and pre-ribosome cleavage and maturation in the nucleoplasm, and they had higher FPKM values in the Gm-Ba and Gm-Ba-Ss than those in the Gm-Ss and Gm treatment. The DEGs relative to metabolic pathways showed similar tendencies in Gm, Gm-Ba and Gm-Ba-Ss and Gm-Ss. The results suggest that *B. amyloliquefaciens* enhances soybean metabolic processes upon infection with *S. sclerotiorum*. Collectively, *B. amyloliquefaciens* could enhance the resistance and metabolism of the soybean for protection against *S. sclerotiorum via* the activation of ribosome and ribosome biogenesis pathways and promote the synthesis of PAL and PPO simultaneously, all processes that are closely related to plant disease resistance.

### *Sclerotinia sclerotiorum* impaired metabolism in soybean and induced senescence *via* the ethylene signal transduction pathway

*Sclerotinia sclerotiorum* can secrete oxalic acid (OA), considered an important toxin in the pathogenic process by inducing host programmed cell death (Kim et al., 2008). OA accumulates at the infection site of *S. sclerotiorum*, causing the pH at the infection site to decrease, thereby improving the activity of cell wall-degrading enzymes, which contributes to *S. sclerotiorum* infection (Guimarães and Stotz, 2004; Hegedus and Rimmer, 2005; Dong et al., 2008; Kim et al., 2008). OA can chelate Ca^2+^ in the plant cell wall at the front of the hypha, which is conducive to the hydrolysis of pectin by polygalacturonase, thus destroying the integrity of the cell wall. The synergistic effect of OA and endogenous polygalacturonase is considered necessary for the complete toxicity of *S. sclerotiorum* (Dong et al., 2008; Kim et al., 2008). Mutations in oxalate synthesis genes in *S. sclerotiorum* significantly decrease its infectivity (Guimarães and Stotz, 2004). In *Arabidopsis*, AAE3 encodes oxalyl-CoA synthetase and is involved in the oxalate degradation process. Thus, AAE3 is expected to contribute to defense against oxalate-producing fungal pathogens (Foster et al., 2012). In our study, three DEGs encoded oxalyl-CoA synthetase and four DEGs encoding PUMP4/5 were identified. These DEGs may be important candidate genes involved in defense against *S. sclerotiorum* in soybean. In Gm-Ss vs. Gm, 19983 DEGs were involved in 21 KEGG pathways, with 19 of these being metabolic pathways. In Gm-Ss vs. Gm, most DEGs were down-regulated, and the most significantly enriched KEGG pathways included biosynthesis of secondary metabolites, metabolic pathways, microbial metabolism in diverse environments, starch and sucrose metabolism, and plant hormone signal transmission, suggesting that *S. sclerotiorum* inoculation had a serious negative effect on the metabolism of soybean seedlings. We identified a set of DEGs encoding SPS, pckA, and pgm, key enzymes in sucrose biosynthesis and glycolysis/gluconeogenesis; their down-regulation in Gm-Ss may have impaired sugar, protein, and nucleic acid metabolism. Based on GO enrichment results, the down-regulated DEGs were mainly associated with molecular functions, such as cytoskeletal protein binding, microtubule binding, tubulin binding, microtubule motor activity, motor activity, and chlorophyll binding, suggesting that *S. sclerotiorum* significantly inhibits the metabolism of soybeans and damages the cytoskeleton and chlorophyll. In Gm-Ss vs. Gm, ethylene and SA signal transduction tended to be activated by the up-regulation of vital DEGs encoding TGA, PR1, ETR, CTR1, and EBF1 (Figure 6A), while jasmonic acid (JA) signal transduction tended to be inactivated by the down-regulation of JAR1, COI1, and MYC2 (Figure 6B). We also identified 117 up-regulated DEGs related to senescence and 43 up-regulated DEGs related to programmed cell death. Moellers et al. (2017) studied the genes related to soybean resistance to *S. sclerotiorum* by genome-wide association and epigenetic studies, and identified candidate genes related to the cell wall structure, sugar allocation, and plant hormone signal transduction as important factors. We analyzed the gene expression patterns of these candidate genes in the four treatments (Gm, Gm-Ba, Gm-Ba-Ss, and Gm-Ss) and found that among the 58 candidate genes proposed, 31 genes were not expressed in all four treatments. The top 20 candidate genes with the highest FPKM values are listed in Supplementary Table 21. If genes with low FPKM values and genes with no significant difference in FPKM values between treatments are filtered out, three up-regulated genes in Gm-Ss vs. Gm are retained, including glyma.11g12900, glyma.07g135400, and glyma.14g188400, which encode aspartyl protease, leucine-rich repeat receptor-like protein kinases (LRR-RLK), and PRs, respectively. We speculated that LRR-RLK and PRs play roles in promoting soybean resistance to *S. sclerotiorum*. Taken together, when *S. sclerotiorum* infects soybean seedlings, the toxin produced by *S. sclerotiorum* destroys the plant cell wall and cytoskeleton and activates the ethylene and salicylic acid signal transduction pathways. The activation of the SA pathway induces the up-regulation of a series of PR genes to resist *S. sclerotiorum* infection. The activation of important components of ethylene signal transduction pathways further activates OA degradation and transport pathways, causing OA accumulation and the disruption of carbohydrate metabolism and secondary metabolism, as well as the activation of genes related to senescence and programmed death, leading to stem necrosis and decay.

In summary, through the analysis of dual transcriptomic sequence data, soybean stem necrosis and decay were found to be induced by *S. sclerotiorum via* the ethylene signal transduction pathway, as well as through activation of genes related to senescence and programmed death. SAR of soybean seedlings to *S. sclerotiorum* can be induced by *B. amyloliquefaciens*. These insights improve our understanding of the antifungal mechanism of soybean and *B. amyloliquefaciens*. Large-scale data obtained from dual RNA-seq have shed some light on the major roles played by the members of the microbiota, with is highly relevant with respect to soybean immune responses. However, the specific mechanisms and key elements of respective interaction networks require further verification using other high-throughput techniques such as yeast two-hybrid, ChIP-Seq, and Chip-chip assays but also small-scale experiments such as construction of knockout models, *in situ* hybridization, and immunohistochemical localization.

## Data availability statement

The datasets presented in this study can be found in online repositories. The names of the repository/repositories and accession number(s) can be found below: BioProject, accession number: PRJNA824616.

## Author contributions

YC contributed to study conception and design, collection and/or assembly of data, and data analysis and interpretation. JL contributed to writing the manuscript. XH, HH, XZ, JG, and JB prepared samples. All authors have read and approved the manuscript.

## Footnotes

1
https://cutadapt.readthedocs.io/en/v2.7/

2
https://ftp.ncbi.nlm.nih.gov/genomes/all/GCF/000/242/855/GCF_000242855.2_ASM24285v2

3
https://ftp.ncbi.nlm.nih.gov/genomes/all/GCF/000/146/945/GCF_000146945.2_ASM14694v2/

4
https://www.soybase.org/GlycineBlastPages/blast_descriptions.php#Wm82.a4.v1.genome.nt

5
http://pathways.embl.de

## Glossary

AAE3: oxalate-CoA ligase
CCoAOMT: caffeoyl-CoA3-*O*-methyltransferase
CESA2: cellulose synthase A catalytic subunit 2
CGMCC: China General Microbiological Culture Collection Center
COI1: coronatine-insensitive protein 1
CSLE1: cellulose synthase-like protein E1
CTR1: serine/threonine-protein kinase CTR1
DEGs: differentially expressed genes
DGE: digital gene expression
DLO1: DMR6-LIKE OXYGENASE 1
DRRG49-C: disease resistance response protein DRRG49-C
EBF1: EIN3-binding F-box protein
ERF1: ethylene-responsive transcription factor 1
ETI: effector-triggered immunity
ETR: ethylene receptor
FDR: false discovery rate
FPKM: fragments per kilobase of transcript per million fragments mapped
GATL2: galacturonosyltransferase-like 2
GATL9: galacturonosyltransferase-like 9
Gm: scratch treatments were used as the control
Gm-Ss: the stems of potted soybean seedlings were inoculated with *S. sclerotiorum*
Gm-Ba: the stems of potted soybean seedlings were inoculated with *B. amyloliquefaciens*
Gm-Ba-Ss: the stems of potted soybean seedlings were inoculated with *S. sclerotiorum* and *B. amyloliquefaciens*
GO: gene ontology
HXK1: Hexokinase-1
ISR: induced systemic resistance
JA: jasmonic acid
JAR1: jasmonic acid-amino synthetase
KEGG: Kyoto Encyclopedia of Genes and Genomes
LRR-RLK: leucine-rich repeat receptor-like protein kinases
MAPKKK18: mitogen-activated protein kinase kinase kinase 18
AMC1: metacaspase-1
MYC2: transcription factor MYC2
NHL10: NDR1/HIN1-like protein 10
OA: oxalic acid
PAL: phenylalanine ammonia lyase
PAMP: pathogen-associated molecular pattern
pckA: phosphoenolpyruvate carboxykinase (ATP)
PDA: potato dextrose agar
pgm: phosphoglucomutase
PPO: polyphenol oxidase
PR1: pathogenesis related protein 1
PRs: pathogenesis-related proteins
PTI: pathogen-associated molecular pattern-triggered immunity
PTI5: pathogenesis-related genes transcriptional activator PTI5
PUMP: mitochondrial uncoupling protein
RPL10: ribosomal protein L10
RPL18A: ribosomal protein L18a
RPL4: ribosomal protein L4
SA: salicylic acid
SAM22: stress-induced protein SAM22
SAR: systemic acquired resistance
SOD: superoxide dismutase
SPS: sucrose-phosphate synthase
SRG1: protein SRG1
STH-2: pathogenesis-related protein STH-2
TGA: transcription factor TGA
UTP22: U3 small nucleolar RNA-associated protein 22
UTP5: U3 small nucleolar RNA-associated protein 5
w82: *Glycine max* cultivar Williams 82
WRKY22: WRKY transcription factor 22
XTH: Xyloglucan endotransglucosylase/hydrolase protein.
