# Supplementary material for: Digital gene expression profiling of the transcriptional response to Sclerotinia sclerotiorum and its antagonistic bacterium Bacillus amyloliquefaciens in soybean
Source: Front Microbiol. 2022 Nov 2;13:1025771. doi: 10.3389/fmicb.2022.1025771 (PMC9666723; doi:10.3389/fmicb.2022.1025771)
Supplement: Supplementary file 1 [file Data_Sheet_1.ZIP › ╕╜╝■1-8/Table S1 Primers sequence information used in qRT-PCR.docx]

| Gene ID | Length (bp) | Primer sequences | Annotation |
| --- | --- | --- | --- |
| Glyma.19G145700 | 133 | F: 5' CCCTGACAGTGGCAATACCG 3'  R: 5' GGCCGCTTGTACTGTTGTGAAAT 3' | Probable pectinesterase, PME8 |
| Glyma.11G101200 | 89 | F: 5' GCCTTCATTTTCACGATTGTTTT 3'  R: 5' CGCAGTCTTTTCGCTCTCG 3' | Lignin-forming anionic peroxidase |
| Glyma.18G219200 | 148 | F: 5' GGACGCCGACCCTCTTATTATTG 3'  R: 5' GCCGAGGACTGTGTTGTTGTAGA 3' | Cysteine-rich receptor-like protein kinase 8, CRK8 |
| Glyma.10G009400 | 88 | F: 5' TTGGCCTCCGCATCTTTG 3'  R: 5' CGGCTTTGTTTACGGCTCTT 3' | Peroxidase 5 |
| Glyma.10G251500 | 131 | F: 5' CCCCCATACGACCTCCAATC 3'  R: 5' CCGGCTCCGACGATTACGA 3' | Thiamine thiazole synthase 2, THI1-2 |
| Glyma.16G165200 | 144 | F: 5’TGAGAAAAACAGCCTCCAAG3’  R: 5’CAGCAGTGTCCCAACCATAG3’ | Chlorophyll a-b binding protein 3, CAB3 |
| Glyma.02g091900 | 95 | F: 5'GACCTTCAACACCCCTGCT3'  R:5'CACCAGAATCCAACACAATA3' | Actin |
